# The effects of breath holding during high‐intensity land‐based ergometry in swimmers

**DOI:** 10.14814/phy2.71004

**Published:** 2026-07-03

**Authors:** Jeremy Walsh, James L. Ramsey, Nasimi A. Guluzade, Robin Faricier, Paul Midgely, Daniel A. Keir, Glen R. Belfry

**Affiliations:** ^1^ School of Kinesiology Western University London Ontario Canada; ^2^ Lawson Health Research Institute London Ontario Canada; ^3^ Toronto General Hospital Research Institute Toronto General Hospital Toronto Ontario Canada

**Keywords:** breath holding, breath‐holding response, competitive swimmers, gas exchange, high‐intensity arm and leg ergometry, muscle deoxygenation

## Abstract

The aim of this study was to compare cardiorespiratory responses during high‐intensity land‐based exercise between free‐breathing and breath‐holding conditions in swimmers. Twenty varsity swimmers (20 ± 2 years, 10 females) performed 20 s bouts of simultaneous arm and leg ergometry exercise at high intensity (total power output = 606 ± 76 W). Heart rate (HR), muscle deoxygenation ([HHb]) and total hemoglobin ([THb]) at the triceps and vastus lateralis, and gas exchange and ventilatory variables were recorded continuously. Blood pressure and lactate were measured pre‐ and post‐exercise. Exercise HR was higher under the breath‐holding condition (free‐breathing [FB] = 118 ± 14 bpm, breath‐holding [BH] = 131 ± 17 bpm; *p* = 0.001) as were post‐exercise blood pressures (systolic: FB = 141 ± 14 mmHg, BH = 156 ± 16 mmHg, *p* = 0.002; diastolic: FB = 71 ± 14 mmHg, BH = 79 ± 9 mmHg; *p* = 0.01, respectively). During exercise, muscle [THb] was higher under the breath‐holding condition (FB = −1.6 ± 2.7 μM, BH = −0.3 ± 1.6 μM; *p* = 0.01). No between‐condition differences in post‐exercise blood lactate (FB = 8.8 ± 1.8 mmol.L^−1^, BH = 9.2 ± 3.9 mmol.L^−1^; *p* = 0.63) or [HHb] (FB = 2.9 ± 2.6 μM, BH = 3.4 ± 2.2 μM; *p* = 0.39) were observed. These data suggest that the exercise response prevailed over the breath‐holding response during high‐intensity land‐based exercise in swimmers and that intrinsic oxygen stores may have been sufficient to sustain the aerobic energy contribution during 20 s high‐intensity exercise while breath holding.

## 1 INTRODUCTION

Breath holding is regularly necessitated during competitive swimming due to the submerged portions that follow the initial dive entry into the pool and during flip turns and the push off the wall, when the swimmer is allowed to travel underwater for up to 15 m (~5 s) per lap (Veiga & Roig, 2016). Furthermore, many elite swimmers do not take any breaths during 50 m sprint freestyle races, which take just over 20 s to complete, to avoid the adverse effects on drag and stroke mechanics imparted by turning the head to breathe (McCabe et al., 2015; Zamparo et al., 2020). Although the energy required for 50 m sprint swimming is provided mainly by anaerobic systems (Pyne & Sharp, 2014), aerobic energy systems contribute approximately 15% of the total energy needs (Capelli et al., 1998; Péronnet & Thibault, 1989). As breath holding prevents inspiration of oxygen (O_2_) to the lung, the O_2_‐dependent aerobic energy contribution to swimming may be compromised by breath holding, and performance impaired (Nishiyasu et al., 2012), unless mitigated by increased reliance on intrinsic O_2_ stores or increased anaerobic energy contributions (Ferretti, 2001; Lindholm & Linnarsson, 2002). However, as the intrinsic O_2_ stores in the lung, dissolved in plasma, and bound to hemoglobin in blood and myoglobin in muscle are not replenished during breath holding (Panneton, 2013), breath holding during exercise may lead to reduced O_2_ availability at the level of the muscle (Ichinose et al., 2018).

This reduced O_2_ availability during breath holding shows a characteristic pattern of respiratory, cardiac and vascular responses triggered by the cessation of breathing (Gooden, 1994), including bradycardia, reduced cardiac output, peripheral vasoconstriction, and increased blood pressure (BP). These responses collectively conserve the limited intrinsic O_2_ stores during breath holding and redistribute O_2_‐rich blood flow from the muscles to the vital organs (Bain et al., 2018; Foster & Sheel, 2005). When breath holding occurs during swimming immersion, the diving response, which is similar to the breath‐holding response (bradycardia and peripheral vasoconstriction), although of greater magnitude, is in opposition to the exercise response (tachycardia and peripheral vasodilation) which emerges simultaneously (Wein et al., 2007). The breath‐holding response has been shown to prevail over the exercise response during low‐intensity exercise (Walsh & Belfry, 2026), whereas the exercise response prevailed over the breath‐holding response during 20 s of high‐intensity exercise (Walsh et al., 2025). In these participants, naïve to breath holding, exercise tachycardia ensured adequate O_2_ delivery so that increased muscle deoxygenation or anaerobic glycolysis did not materialize, suggesting that intrinsic O_2_ stores may have been sufficient to meet the aerobic energy demand (Walsh et al., 2025).

However, a stronger breath‐holding response has been reported in those who are habituated to breath holding, for example, experienced breath‐hold divers show greater reductions in heart rate (HR), peripheral blood flow, and greater increases in systolic and diastolic BP, compared to untrained control participants (Bain et al., 2018; Joulia et al., 2002; Joulia et al., 2009). Furthermore, when participants naïve to breath holding performed daily repeated maximal duration breath holds at rest, there was an enhanced breath‐holding response within 2 weeks, that is., an earlier onset and more pronounced bradycardia, a greater rise in BP, and reduced arterial O_2_ desaturation (Engan et al., 2013; Joulia et al., 2003; Schagatay et al., 2000). Collectively, these findings are indicative of increased O_2_‐conserving efficiency and suggest that habitual breath holding results in an enhanced breath‐holding response (Engan et al., 2013; Joulia et al., 2002; Joulia et al., 2009). While these reports have involved maximal‐duration breath holds, artistic swimmers who perform frequent breath holds in training and competition, have also demonstrated an enhanced breath‐holding response at rest and during low‐intensity exercise, as shown by longer breath‐hold times and greater bradycardia, compared to controls (Alentejano et al., 2012). Whether the frequent short‐duration breath holds performed by competitive swimmers over years of training and racing may result in an enhanced breath‐holding response is unknown, considering that swimmers have been shown to have a longer maximal breath‐holding durations and reduced hypoxic ventilatory responses, but higher HR during breath holding, compared to controls (Arce‐Álvarez et al., 2021). If this habituation does indeed lead to an enhanced breath‐holding response in swimmers, the breath‐holding response might prevail over the exercise response and the aerobic contribution to energy production may be compromised, contrary to the findings in nonswimmers performing an identical exercise protocol (Walsh et al., 2025). Conversely, there is the possibility that adaptations associated with the frequent breath holding while training and racing by competitive swimmers may result in an adaptive exercise response that overrides the breath‐holding response and the aerobic contribution to energy production may not be compromised, as was shown in nonswimmers (Walsh et al., 2025).

The objectives of this study were to determine (1) the effects of breath holding during high‐intensity land‐based exercise on HR, BP, muscle oxygenation, blood lactate concentration ([La^−^]) and gas exchange and ventilatory variables in competitive swimmers, and (2) to establish whether the exercise response would prevail over the breath‐holding response by comparing 20 s land‐based high‐intensity arm and leg ergometry exercise under free‐breathing and breath‐holding conditions. These land‐based exercise modalities ensured that the specific breath‐holding responses would not be confounded by the diving response that is associated with water immersion.

## 2 MATERIALS AND METHODS

### 2.1 Participants

Twenty competitive swimmers (mean ± standard deviation (SD) age: 20 ± 2 years, height: 179 ± 10 cm, weight: 74 ± 9 kg, 10 females) volunteered to participate in this study (Table 1). All participants were members of the local University's Varsity Swim team; one was a Canadian University Championship medalist, and four were Ontario Provincial University medalists. All swimmers had a minimum of 8 years of competitive swimming experience, were nonsmokers, had no history of cardiorespiratory disease, and were not taking any medications that might influence cardiorespiratory or metabolic responses to exercise. All participants provided written informed consent, and all procedures were in accordance with the 1964 Helsinki declaration and its later amendments or comparable ethical standards and were approved by the Western University Research Ethics Board for Health Sciences Research Involving Human Participants (REB #:118179).

**TABLE 1:** Participant characteristics and power outputs used during 20 s of simultaneous arm and leg ergometry exercise.

Sex	Participant	Age	Height	Mass	Power output (W)		
		(years)	(cm)	(kg)	Legs	Arms	Total
Males	1	19	188	82	482	193	675
	2	20	183	93	547	219	766
	3	24	188	80	471	188	659
	4	19	194	86	506	202	708
	5	20	182	77	453	181	634
	6	18	193	81	476	191	667
	7	18	180	70	412	165	576
	8	20	193	77	453	181	634
	9	22	175	74	435	174	609
	10	22	183	79	465	186	651
	Mean	20	186	80	470	188	658
	SD	2	6	6	37	15	52
Females	1	21	168	66	388	155	544
	2	18	180	77	453	181	634
	3	21	180	76	447	179	626
	4	19	168	68	400	160	560
	5	19	180	75	441	176	618
	6	21	163	59	347	139	486
	7	19	177	68	400	160	560
	8	19	170	60	353	141	494
	9	23	168	66	388	155	544
	10	21	160	57	335	134	469
	Mean	20	171	67	395	158	553
	SD	2	7	7	42	17	59

Abbreviations: cm, centimeters; kg, kilograms; SD, standard deviation; W, watts.

### 2.2 Protocol

This study used an identical procedure to previously described (Walsh et al., 2025). Simultaneous arm and leg ergometry were used to mimic the muscular demands of swimming. Leg ergometry was performed using an electromagnetically braked cycle ergometer (Velotron; RacerMate, Seattle, WA) and arm ergometry was performed using an adapted cycle ergometer (Lode Corival 400; Groningen, Netherlands). Subjects attended a familiarization session at least 48 h prior to testing, in which they simultaneously pedaled and arm cranked under free‐breathing and breath‐holding conditions. Wingate Anaerobic Test parameters were used to ensure participants were exercising at a high intensity: power output (PO) for the arms was calculated as 0.04 kg per kg body weight (BW) at a cadence of 60 revolutions per minute (rpm) (Forbes et al., 2014) and 0.075 kg per kg at a cadence of 80 for the legs (Zupan et al., 2009). The mean ± SD PO used for the arms and legs were 173 ± 22 Watts (W) and 433 ± 55 W, respectively, for a total PO of 606 ± 76 W (Table 1).

Participants were tested twice, with a minimum of 48 h between sessions, as described previously (Walsh et al., 2025). Participants abstained from eating for 2 h prior to testing and strenuous exercise, recreational drug use, alcohol, and caffeine for ≥12 h before each test, and all tests were scheduled at the same time of day (within ±1 h). In brief, on one occasion the exercise was performed while breath holding and on the other while free breathing, and the order of testing was randomized. Participants were advised whether they would be exercising under free‐breathing or breath‐holding conditions prior to each test, and for breath holding, they were instructed to take their last breath during the 3 s countdown to the start of exercise and to not breathe again until the end of the exercise bout. At the onset of exercise, participants pedaled the legs and cranked the arms for 20 s. At the end of the exercise bout, participants remained seated on the cycle ergometer, with their legs resting on the stools either side of the pedals and their hands resting on their knees for 15 min.

### 2.3 Measurements

#### 2.3.1 Heart rate and blood pressure

Heart rate was measured by a chest strap monitor (Polar H10, Polar Electro Oy, Kempele, Finland) and recorded on a second‐by‐second basis by a Polar Beat application (version 3.5.6., Polar Electro Oy, Kempele, Finland). Systolic and diastolic BP were measured using an automatic sphygmomanometer (Patient Monitor, PM80D, Guandong, China) 4 min pre‐ and immediately post‐exercise.

#### 2.3.2 Muscle oxygenation

Muscle oxygenation was monitored continuously using near infrared spectroscopy (NIRS) (Oxiplex TS, model 95,205, ISS, Champaign, IL) as described previously (Walsh et al., 2025). In brief, NIRS probes were placed on the left vastus lateralis (midway between the lateral epicondyle and greater trochanter of the femur) and triceps brachii (midway between the acromion of the scapula and the olecranon of the ulna) muscles. An elastic strap secured each probe in place. An optically dense, black vinyl sheet was placed over each probe to prevent exposure to extraneous light. The thigh and arm probes were wrapped with elastic bandages to further minimize the intrusion of extraneous light and movement. NIRS measurements were collected second‐by‐second from 3 min pre‐exercise to 15 min post‐exercise. [HHb] and oxygenated hemoglobin ([HbO_2_]) concentrations were measured, whereas total hemoglobin concentration ([THb]) and S_m_O_2_ were derived with this apparatus. [THb] was calculated as the sum of [HHb] and [HbO_2_], and S_m_O_2_ as the percentage of [HbO_2_] to [THb].

#### 2.3.3 Lactate

Blood [La^−^] from the left index finger was measured 3 min pre‐ and post‐exercise, and the sample was immediately analyzed by SensLab GmbH Lactate SCOUT (Leipzig, Germany) capillary lactate analyzer.

#### 2.3.4 Gas exchange and ventilatory variables

Oxygen uptake (V̇O_2_), carbon dioxide production (V̇CO_2_), minute ventilation (V̇_E_), and end‐tidal partial pressures of O_2_ (P_et_O_2_) and CO_2_ (P_et_CO_2_) were measured breath‐by‐breath by metabolic cart (Quark, CPET; Cosmed, Rome, Italy). Inspired and expired volume rates were measured by a low‐dead‐space turbine after being calibrated with a syringe of known volume (3 L). Fractional concentrations of inspired and expired O_2_ and CO_2_ for each breath were assessed by gas analyzers that were calibrated before each test using a gas mixture of known concentration. As gas exchange and ventilatory data were not available during breath holding, the 5 s bins immediately pre‐ and post‐exercise were used for V̇O_2_, V̇CO_2_, and V̇_E_, and the final breath pre‐exercise and the first breath post‐exercise were used for P_et_O_2_ and P_et_CO_2_. During high‐intensity exercise, ventilatory buffering results in increased breathing frequencies that reduce alveolar‐capillary diffusion times for O_2_ and CO_2_, giving rise to exaggerated higher and lower end‐tidal pressures, respectively, than that of pulmonary capillary pressures (Whipp, 1977). Since P_et_O_2_ and P_et_CO_2_ during the breath holds of the present study were determined from the last inspiration before the breath hold and the expiration occurring 20 s later, the effects of faster breathing frequencies on the O_2_ and CO_2_ pulmonary arterial and alveolar end‐tidal pressure equilibrium would not evolve (Lim et al., 2018).

### 2.4 Data analyses

Aberrant NIRS data points due to movement artifacts during high‐intensity exercise and gas exchange and ventilatory data points due to coughs, sighs or swallows, identified as those positioned more than 3 SD from the local mean, were removed prior to analysis using OriginPro, version 2023b (OriginLab Corporation, Northampton, MA, USA). Breath‐by‐breath gas exchange and ventilatory data were linearly interpolated on a second‐by‐second basis. NIRS data were adjusted to baseline values and changes in S_m_O_2_, [HHb] and [THb] respective to their baseline values (ΔS_m_O_2_, Δ[HHb] and Δ[THb]) were calculated for the vastus lateralis and triceps muscles. As well as analyzing the individual muscles, data from both muscles were averaged and analyzed as combined whole‐body muscular ΔS_m_O_2_, Δ[HHb] and Δ[THb]. All second‐by‐second data were bin averaged into 5 s, 20 s, and 1 min bins for graphing and statistical purposes.

### 2.5 Statistical analyses

Data are presented as mean ± SD. Paired *t*‐tests were used to determine whether there were any differences in the means of the dependent variables between free‐breathing and breath‐holding conditions at baseline and during exercise. Cohen's *d* was used for effect sizes, interpreted as small (*d* > 0.2), moderate (*d* > 0.5), or large (*d* > 0.8). To determine whether there were any interactions between the main effects of sex, condition (free‐breathing and breath‐holding), and time, three‐way repeated measures analysis of variance (ANOVA) was used. For the time factor, the baseline measure and four 5 s bins of the 20 s exercise period were used for HR and muscle oxygenation variables, and pre‐ and post‐exercise measures were used for BP and [La^−^]. As stated previously, the 5 s bins immediately pre‐ and post‐exercise were used for V̇O_2_, V̇CO_2_, and V̇_E_, and the final breath pre‐exercise and the first breath post‐exercise were used for P_et_O_2_ and P_et_CO_2_. To determine the latent effects of breath holding during exercise, three‐way repeated measures ANOVA was used to determine whether there were any interactions between the main effects of sex, condition, and time, post‐exercise, with 1 min bins for the first 5 min of the recovery period used as the time factor for all variables.

Data were assessed for normality using the Shapiro–Wilk test. Mauchly's test of sphericity was used to determine whether the variances among the differences of all levels of the independent variables were equal. When the assumption of sphericity was violated, the Greenhouse–Geisser correction was used. Effect sizes for ANOVA are presented as partial eta squared ($\eta_{p}^{2}$) and interpreted as small ($\eta_{p}^{2}$ = 0.01), medium ($\eta_{p}^{2}$ = 0.06) or large ($\eta_{p}^{2}$ = 0.14). Pairwise comparisons with Bonferroni corrections were used when significant interaction or main effects were found. All statistical analyses were performed using IBM SPSS Statistics version 29.0.2.0 (IBM Corp., Armonk, N.Y., USA). Statistical significance was accepted at a level of *p* < 0.05.

## 3 RESULTS

At baseline, there were no significant differences between free‐breathing and breath‐holding conditions for any of the variables (Table 2). Exploratory sex analyses found main effects of sex for V̇O_2_, V̇CO_2_, V̇E, P_et_CO_2_, Δ[THb], Δ[HHb], and BP; there were no interactions between the effects of sex, condition and time, or sex and condition for any variables (Table 3). Muscle oxygenation findings were not different between vastus lateralis and triceps brachii muscles, and so the combined muscle oxygenation data are presented here.

**TABLE 2:** Baseline measures of heart rate and gas exchange and ventilatory variables.

	Baseline		*p* Value
	FB	BH	
Heart rate (bpm)	80 ± 11	84 ± 11	0.10
V̇O_2_ (L.min^−1^)	0.45 ± 0.08	0.47 ± 0.09	0.15
V̇CO_2_ (L.min^−1^)	0.40 ± 0.10	0.44 ± 0.09	0.08
V̇_E_ (L.min^−1^)	14 ± 2.7	15 ± 2.8	0.18
P_et_O_2_ (mmHg)	109 ± 4	109 ± 4	0.89
P_et_CO_2_ (mmHg)	33 ± 3	34 ± 3	0.06

*Note*: Data presented are mean ± SD of the 180 s baseline period. *n* = 20.

Abbreviations: BH, breath‐holding; bpm, beats per minute; FB, free‐breathing; L.min^−1^, liters per minute; mmHg, millimeters of mercury; P_et_CO_2_, end‐tidal partial pressure of carbon‐dioxide; P_et_O_2_, end‐tidal partial pressure of oxygen; V̇CO_2_, rate of carbon dioxide production; V̇_E_, minute ventilation; V̇O_2_, rate of oxygen uptake.

**TABLE 3:** Main effects of sex and interactions between the effects of sex, condition and time.

	Main effect of sex			Sex × condition × time interaction
	Males	Females	*p*	*p*
Exercise				
Heart rate (bpm)	117 ± 13	115 ± 11	0.69	0.74
∆S_m_O_2_ (%)	−5.7 ± 0.9	−4.9 ± 1.9	0.24	0.84
∆[THb] (μM)	−0.7 ± 2.0	−0.8 ± 0.9	0.53	0.71
∆[HHb] (μM)	3.9 ± 1.0	1.2 ± 0.8	<0.001^a^	0.81
V̇O_2_ (L.min^−1^)	2.55 ± 0.3	2.06 ± 0.3	0.003^a^	0.14
V̇CO_2_ (L.min^−1^)	2.17 ± 0.4	1.65 ± 0.3	0.001^a^	0.19
V̇_E_ (L.min^−1^)	64 ± 10	49 ± 8	0.002^a^	0.16
P_et_O_2_ (mmHg)	96 ± 6	100 ± 5	0.14	0.27
P_et_CO_2_ (mmHg)	41 ± 3	38 ± 2	0.02^a^	0.15
Systolic BP (mmHg)	145 ± 7	132 ± 6	0.005^a^	0.88
Diastolic BP (mmHg)	77 ± 8	69 ± 5	0.01^a^	0.43
Lactate (mmol.L^−1^)	5.7 ± 1.5	5.3 ± 1.0	0.48	0.88
Recovery				
HR (bpm)	109 ± 16	101 ± 13	0.21	0.16
∆S_m_O_2_ (%)	1.5 ± 2.1	1.5 ± 1.2	0.93	0.54
∆[THb] (μM)	5.5 ± 2.5	1.8 ± 1.2	<0.001^a^	0.59
∆[HHb] (μM)	−0.1 ± 1.3	0.1 ± 0.6	0.72	0.64
V̇O_2_ (L.min^−1^)	1.29 ± 0.2	0.90 ± 0.1	<0.001^a^	0.21
V̇CO_2_ (L.min^−1^)	1.79 ± 0.4	1.20 ± 0.1	<0.001^a^	0.15
V̇_E_ (L.min^−1^)	52 ± 10	38 ± 4	<0.001^a^	0.60

*Note*: Data presented are mean ± SD. *n* = 10 males, *n* = 10 females.

Abbreviations: [HHb], deoxygenated hemoglobin; [THb], total hemoglobin; BP, blood pressure; bpm, beats per minute; L.min^−1^, liters per minute; mmHg, millimeters of mercury; mmol.L^−1^, millimoles per liter; P_et_CO_2_, end‐tidal partial pressure of carbon‐dioxide; P_et_O_2_, end‐tidal partial pressure of oxygen; S_m_O_2_, muscle oxygen saturation; V̇CO_2_, minute rate of carbon dioxide production; V̇_E_, minute ventilation; V̇O_2_, minute rate of oxygen uptake; Δ, change from baseline; μM, micromoles.

^a^ Significant difference between males and females.

### 3.1 Exercise

#### 3.1.1 Heart rate

**FIGURE 1:**
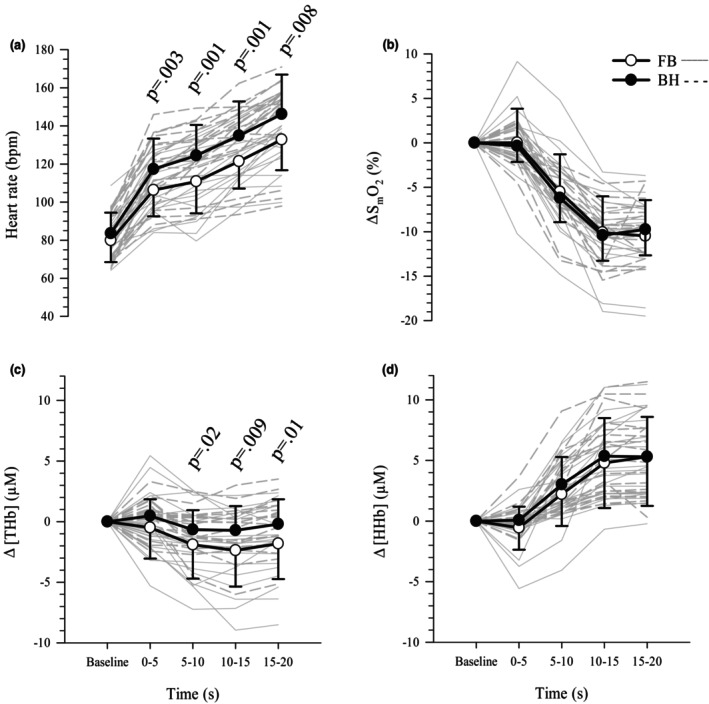
Heart rate and muscle oxygenation during exercise. Data presented are mean (symbols) ± standard deviation (error bars) for the baseline period and in 5 s bins for the 20 s exercise period for (a) heart rate, and changes from baseline in (b) muscle oxygen saturation (ΔS_m_O_2_), (c) total hemoglobin (Δ[THb]), and (d) deoxygenated hemoglobin (Δ[HHb]). Gray lines represent individual responses. *n* = 20 for all panels. μM, micromoles; BH, breath holding (black circles); bpm, beats per minute; FB, free breathing (white circles).

During exercise, HR was significantly higher under the breath‐holding condition (free‐breathing [FB] = 118 ± 14 bpm, breath‐holding [BH] = 131 ± 17 bpm; *p* = 0.001, *d* = 0.83). There was a significant interaction between the effects of condition and time on HR (*p* = 0.04, $\eta_{p}^{2}$ = 0.16), which was not significantly different between conditions at baseline but was significantly higher under the breath‐holding condition for each 5 s bin of the exercise period (Figure 1a).

#### 3.1.2 Blood pressure

**FIGURE 2:**
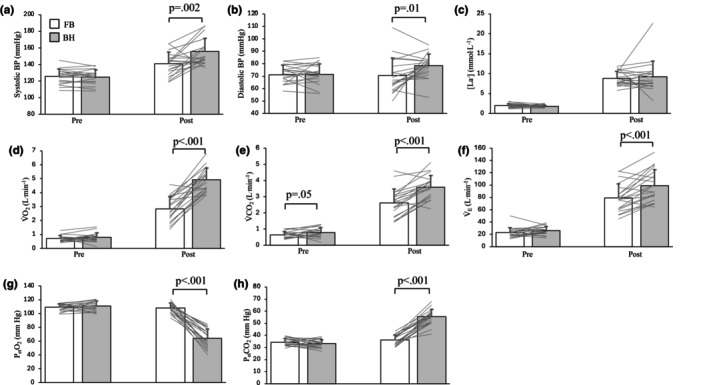
Blood pressure, lactate, and gas exchange and ventilatory data. Data presented are mean (bars) ± standard deviation (error bars) of pre‐ and post‐exercise measures for (a) systolic and (b) diastolic blood pressures (BP), and (c) blood lactate; the 5 s immediately pre‐ and post‐exercise for (d) rate of oxygen uptake (V̇O_2_), (e) rate of carbon dioxide production (V̇CO_2_), and (f) minute ventilation (V̇_E_); and the final breath pre‐ and first breath post‐exercise for end‐tidal partial pressures of (g) oxygen (P_et_O_2_) and (h) carbon‐dioxide (P_et_CO_2_). Lines represent individual responses. *n* = 20 for all panels. BH, breath holding (graygrey bars); FB, free breathing (white bars); L.min^−1^, liters per minute; mmHg, millimeters of mercury; mmol.L^−1^, millimols per liter; Post, post‐exercise; Pre, pre‐exercise.

Systolic and diastolic BP (Figure 2a,b) were not significantly different between free‐breathing and breath‐holding conditions pre‐exercise (systolic: FB = 126 ± 9 mmHg, BH = 125 ± 9 mmHg; *p* = 0.57, diastolic: FB = 71 ± 8 mmHg, BH = 71 ± 9 mmHg; *p* = 0.95) but were significantly higher under the breath‐holding condition post‐exercise (systolic: FB = 141 ± 14 mmHg, BH = 156 ± 16 mmHg; *p* = 0.002; diastolic: FB = 71 ± 14 mmHg, BH = 79 ± 9 mmHg; *p* = 0.01).

#### 3.1.3 Muscle oxygenation

There were no significant differences between free‐breathing and breath‐holding conditions during exercise in ΔS_m_O_2_ (FB = −7 ± 4%, BH = −7 ± 2%; *p* = 0.87, *d* = 0.04, Figure 1b), or Δ[HHb] (FB = 2.9 ± 2.6 μM, BH = 3.4 ± 2.2 μM; *p* = 0.39, *d* = 0.20, Figure 1d). Δ[THb] was significantly higher under the breath‐holding condition during exercise (FB = −1.6 ± 2.7 μM, BH = −0.3 ± 1.6 μM; *p* = 0.01, *d* = 0.61). There was a significant interaction between the effects of condition and time on Δ[THb], which was not different between conditions from 0 to 5 s of exercise but was higher under the breath‐holding condition from 5 to 20 s of exercise (*p* = 0.01, $\eta_{p}^{2}$ = 0.21; Figure 1c).

#### 3.1.4 Lactate

There were no significant differences in [La^−^] between free‐breathing and breath‐holding conditions pre‐exercise (FB = 2.0 ± 0.5, BH = 1.8 ± 0.29; *p* = 0.20) or post‐exercise (FB = 8.8 ± 1.8 mmol.L^−1^, BH = 9.2 ± 3.9 mmol.L^−1^; *p* = 0.63) (Figure 2c).

#### 3.1.5 Gas exchange and ventilatory variables

V̇O_2_ was not significantly different between free‐breathing and breath‐holding conditions pre‐exercise (FB = 0.69 ± 0.23 L.min^−1^, BH = 0.78 ± 0.32 L.min^−1^; *p* = 0.20) but was significantly higher under the breath‐holding condition post‐exercise (FB = 2.82 ± 0.91 L.min^−1^, BH = 4.91 ± 0.84 L.min^−1^; *p* < 0.001) (Figure 2d). V̇CO_2_ was significantly higher under the breath‐holding condition immediately pre‐exercise (FB = 0.64 ± 0.20 L.min^−1^, BH = 0.78 ± 0.29 L.min^−1^; *p* = 0.05) and post‐exercise (FB = 2.62 ± 0.86 L.min^−1^, BH = 3.59 ± 0.72 L.min^−1^; *p* < 0.001) (Figure 2e). V̇_E_ was not significantly different between free‐breathing and breath‐holding conditions pre‐exercise (FB = 23 ± 8 L.min^−1^, BH = 26 ± 7 L.min^−1^; *p* = 0.07) but was significantly higher under the breath‐holding condition post‐exercise (FB = 79 ± 23 L.min^−1^, BH = 99 ± 26 L.min^−1^; *p* < 0.001) (Figure 2f).

P_et_O_2_ and P_et_CO_2_ were not significantly different between free‐breathing and breath‐holding conditions pre‐exercise (P_et_O_2_: FB = 109 ± 5 mmHg, BH = 111 ± 7 mmHg; *p* = 0.28; P_et_CO_2_: FB = 34 ± 3 mmHg, BH = 33 ± 3 mmHg; *p* = 0.14) but P_et_O_2_ was significantly lower (FB = 108 ± 7 mmHg, BH = 64 ± 14 mmHg; *p* < 0.001, Figure 2g) and P_et_CO_2_ was significantly higher (FB = 36 ± 4 mmHg, BH = 55 ± 6 mmHg; *p* < 0.001, Figure 2h) under the breath‐holding condition, post‐exercise.

### 3.2 Recovery

**FIGURE 3:**
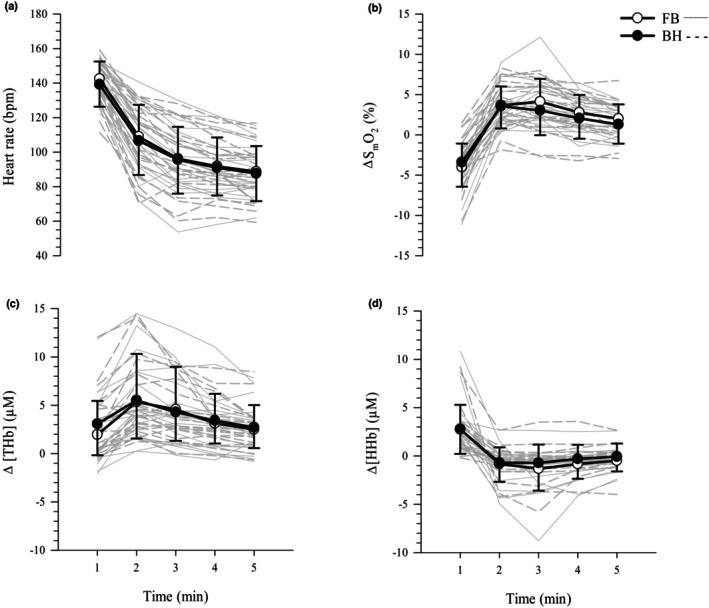
Heart rate and muscle oxygenation during recovery. Data presented are mean (symbols) ± standard deviation (error bars) of the 1 min bins of the first 5 min post exercise for (a) heart rate, and changes from baseline in (b) muscle oxygen saturation (ΔSmO_2_), (c) total hemoglobin (Δ[THb]), and (d) deoxygenated hemoglobin (Δ[HHb]). Gray lines represent individual responses. *n* = 20 for all panels. μM, micromoles; BH, breath holding (black circles); bpm, beats per minute; FB, free breathing (white circles).

**FIGURE 4:**
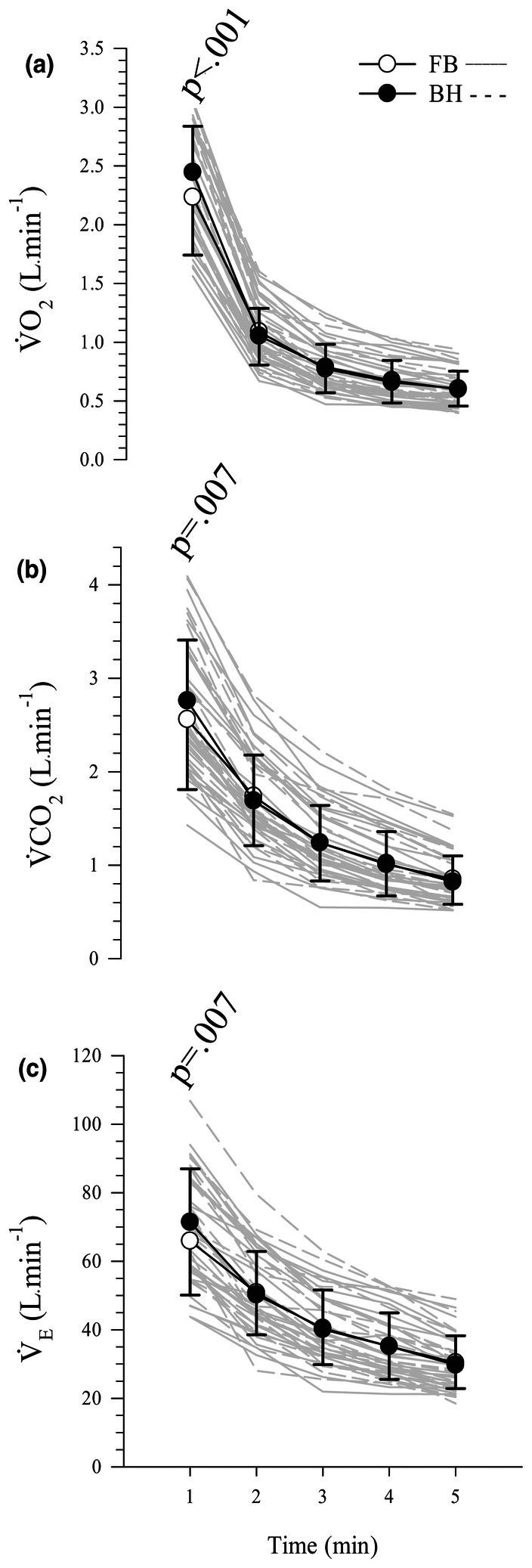
Gas exchange and ventilatory data during recovery. Data presented are mean (symbols) ± standard deviation (error bars) of the 1 min bins of the first 5 min post exercise for A) rate of oxygen uptake (V̇O_2_), B) rate of carbon dioxide production (V̇CO_2_), and C) minute ventilation V̇_E_. Gray lines represent individual responses. *n* = 20 for all panels. BH, breath holding (black circles); FB, free breathing (white circles); L.min^−1^, liters per minute.

There were no significant differences in HR (*p* = 0.34, $\eta_{p}^{2}$ = 0.06, Figure 3a), ΔS_m_O_2_ (*p* = 0.11, $\eta_{p}^{2}$ = 0.12; Figure 3b), Δ[THb] (*p* = 0.16, $\eta_{p}^{2}$ = 0.10; Figure 3c), or Δ[HHb] (*p* = 0.41, $\eta_{p}^{2}$ = 0.04; Figure 3d) between conditions during the first 5 min of recovery post‐exercise. There were significant interactions between the effects of condition and time on V̇O_2_ (*p* < 0.001, $\eta_{p}^{2}$ = 0.62), V̇CO_2_ (*p* < 0.001, $\eta_{p}^{2}$ = 0.36), and V̇_E_ (*p* < 0.001, $\eta_{p}^{2}$ = 0.32) which were all significantly higher under the breath‐holding condition for the first min of recovery (V̇O_2_: FB = 2.24 ± 0.49 L.min^−1^, BH = 2.45 ± 0.39 L.min^−1^, *p* < 0.001; V̇CO_2_: FB = 2.56 ± 0.75 L.min^−1^, BH = 2.76 ± 0.65 L.min^−1^, *p* = 0.007, V̇_E_: FB = 66 ± 16 L.min^−1^, BH = 71 ± 16 L.min^−1^; *p* = 0.007), but not different thereafter (Figure 4).

## 4 DISCUSSION

The main findings of this study of breath‐holding during high‐intensity exercise in male and female competitive swimmers were that (1) there was increased HR, Δ[THb], and diastolic BP under the breath‐holding condition, compared to free‐breathing, (2) the exercise response prevailed over the breath‐holding response, and (3) O_2_ availability was not compromised as there was not an increased reliance on muscle deoxygenation or anaerobic glycolysis considering the similar Δ[HHb] and [La^−^] between free‐breathing and breath‐holding.

### 4.1 Physiological response during exercise

#### 4.1.1 Free‐breathing response vs. breath‐holding response

An elevated HR at the initiation of exercise while breath holding (Figure 1a) may be expected due to the sympathetic response to exercise, combined with the stimulatory effects of the inspiration prior to the breath hold (Bouten et al., 2021; Caspers et al., 2011; Wein et al., 2007). Thus, HR increases for the first 5–10 s due to these stimuli, but breath‐holding‐induced bradycardia subsequently prevails due to the reduced activity of pulmonary stretch receptors leading to increased parasympathetic drive (Alboni et al., 2011; Bouten et al., 2021; Lindholm et al., 2002). It is suggested that a subsequent bradycardia was not observed in the current study due to increased and continued sympathetic drive of the high‐intensity exercise, superimposed on the factors responsible for the initial tachycardia, which resulted in the tachycardia being sustained for the entire exercise period. In essence, the exercise response prevailed over the breath‐holding response, similar to what has been observed in a different study of nonswimmers (Walsh et al., 2025).

The increased systolic and diastolic BP immediately post‐exercise under the breath‐holding condition (Figure 2a,b) are evidence of the breath‐holding response. During exercise while free breathing, increased HR leads to increased cardiac output and systolic BP (Fletcher et al., 2001), but the fall in peripheral resistance due to muscle vasodilation results in diastolic BP falling below resting values (Dillon & Joyner, 2025). However, during breath holding, peripheral vasoconstriction from elevated sympathetic nervous activity progressively leads to an increase in both systolic and diastolic BP (Bain et al., 2018; Dujic & Breskovic, 2012; Perini et al., 2008). There was a greater magnitude increase in BP in swimmers in the current study, compared to nonswimmers in a previous study (Walsh et al., 2025), and both systolic and diastolic BP increased in swimmers in the current study, whereas only systolic BP increased in nonswimmers in the previous study (Walsh et al., 2025). It appears that there may have been a stronger response to breath holding during high‐intensity land‐based exercise in swimmers in the current study, compared to nonswimmers in a previous study. The greater BP response in swimmers may have been due to a stronger peripheral vasoconstriction. Although peripheral vasoconstriction and increased BP are both components of the breath‐holding response, the increased HR and ∆[THb] (Figure 1c), (which may reflect increased capillary hematocrit and blood flow (Barstow, 2019; Grassi & Quaresima, 2016), or passive venous congestion considering the concomitant increase in systemic BP), may have counteracted the breath‐holding‐induced vasoconstriction and ensured adequate O_2_ delivery to the muscles. In a comparison of trained divers habituated to breath holding and triathletes naive to breath holding, HR and peripheral blood flow were unchanged in the divers but significantly increased in the triathletes after 60 s of apnea while cycling at 25% maximal power (Joulia et al., 2009). Although the exercise response prevailed over the breath‐holding response in the triathletes, the enhanced breath‐holding response in the divers was sufficient to prevail over the exercise response (Joulia et al., 2009).

Others have suggested that the characteristics of breath‐hold divers that contribute to enhanced O_2_ delivery mechanisms during the breath‐holding response include increased lung volumes and therefore O_2_ storage capacity (Ferretti & Costa, 2003), depressed ventilatory responses to hypercapnia and hypoxia (Ferretti & Costa, 2003; Foster & Sheel, 2005), improved arterial elasticity and compliance (Tanaka et al., 2016), larger spleen volume (Elia, Barlow, et al., 2021; Schagatay et al., 2012), greater red blood cell volume (Baković et al., 2005), and increased myoglobin and hemoglobin concentrations as well as increased capillary density and smaller cross‐sectional muscle fiber area in muscles (Elia, Gennser, et al., 2021). Further, the reduced chemosensitivity to progressive hypoxia and hypercapnia has been observed in breath‐holding athletes and the resultant decrease in respiratory drive increases the magnitude of the cardiovascular responses (Foster & Sheel, 2005). Swimmers have demonstrated some of these features that might contribute to an enhanced breath‐holding response such as greater lung volumes and pulmonary diffusing capacities (Andrew et al., 1972; Cordain & Stager, 1988; Yost et al., 1981) and a reduced hypoxic ventilatory response (Arce‐Álvarez et al., 2021).

In the current study, the increased HR and ∆[THb] during exercise, in the presence of increased systolic and diastolic BP post‐exercise, while breath holding, compared to free breathing, also suggest that the exercise response dominated over the apnea response during 20 s of arm and leg ergometry exercise at a total power output of 606 ± 76 W. The exercise‐induced tachycardia observed in the current study suggests parasympathetic withdrawal and increased sympathetic activity, in contrast to breath‐holding‐induced bradycardia which is due to increased parasympathetic activity (Foster & Sheel, 2005; Wein et al., 2007). When breath holding occurs during exercise, the overall response is determined by the algebraic summation of these conflicting stimuli (Fico et al., 2022). While the exercise response is dependent on exercise intensity—the higher the intensity, the greater the parasympathetic withdrawal and sympathetic input on the pacemaker of the heart, the apneic parasympathetic influence on HR does not depend on exercise intensity, per se (Wein et al., 2007). Rather, the breath‐holding response is dependent on breath‐hold duration—the longer the duration, the greater the response (Elia, Gennser, et al., 2021). Thus, for a given fixed duration breath hold, exercise intensity will determine the overall response, with the breath‐holding response prevailing at lower intensities, but at higher intensities, the exercise response is more likely to prevail (Guimard et al., 2021), as was observed in the current study. Although the breath‐holding response is stronger with longer breath‐hold durations, with higher exercise intensity, the breath‐holding duration will be shorter (Wein et al., 2007). As such, at higher exercise intensities the breath‐holding response may be limited by an inability to sustain the breath hold for long enough for its effects to be observed (Bergman et al., 1972; Guimard et al., 2021; Wein et al., 2007). Furthermore, it has been suggested that a minimum of 30–40 s of breath‐holding is required to obtain the full development of the breath‐holding response bradycardia (Caspers et al., 2011; Jung & Stolle, 1981; Schagatay et al., 1999). In the current study of 20 s of high‐intensity land‐based exercise, the exercise response prevailed over the breath‐holding response in competitive swimmers. This is similar to what was previously found in nonswimmers (Walsh et al., 2025) suggesting that adaptation from years of swimming training may be moot.

### 4.2 Energy production

Energy availability was not compromised by breath holding, considering that identical power outputs were sustained under both breathing conditions. It appears that, despite reduced O_2_ supply from the atmosphere during breath holding, the increased ∆[THb] (Figure 1c) suggests that O_2_ delivery was increased compared to free breathing. Moreover, the unchanged ∆S_m_O_2_ (Figures 1b) and ∆[HHb] (Figure 1d) between free‐breathing and breath‐holding conditions also suggest that O_2_ utilization at the muscles was similar between conditions. Furthermore, there was no increased contribution from anaerobic glycolysis due to breath holding, considering [La^−^] was not different between conditions (Figure 2c). Additionally, our data suggest that O_2_ availability was not compromised at the level of the muscle as there was no increase in muscle deoxygenation. The increased V̇O_2_ (Figure 2d) and decreased P_et_O_2_ (Figure 2g) immediately post‐exercise, compared to free breathing, reflect the use of intrinsic O_2_ stores during the exercise. The excess post‐exercise O_2_ consumption under the breath‐holding condition, compared to free breathing, serves to pay the O_2_ deficit and replenish the intrinsic O_2_ stores used during exercise (Børsheim & Bahr, 2003; Vogiatzis et al., 2007) and the decreased P_et_O_2_ immediately post‐exercise under the breath‐holding condition is indicative of the concomitant reduction in lung and dissolved O_2_ stores in the plasma (Ferretti et al., 1991). These findings collectively suggest that intrinsic O_2_ stores may have been sufficient for the required aerobic phosphorylation of the 20 s exercise bout while breath holding. As no measure of the high‐energy phosphate system was taken in the present study, it is unknown whether there was an increased contribution from these high‐energy stores.

There findings are similar to those in nonswimmers in a previous study (Walsh et al., 2025), for example, tachycardia and unchanged muscle deoxygenation during exercise, no differences in [La^−^], and decreases in P_et_O_2_ and increases in V̇O_2_, post‐exercise. The main differences were that there was increased ∆[THb] in swimmers, which was not observed in nonswimmers, and the aforementioned BP differences, suggesting a swim training induced adaptation. Conversely, the data in the present study suggest that the tachycardia during exercise while breath holding was similar in swimmers and nonswimmers and may have served to maintain adequate O_2_ availability at the muscle by increasing O_2_ delivery. However, the greater increase in O_2_ delivery in swimmers, indicated by the increased ∆[THb], may have been underpinned by an increased stroke volume reflecting the specific cardiac training adaptations from their years of swimming (Wasfy et al., 2019).

### 4.3 Physiological response post‐exercise

There were no differences in HR or muscle oxygenation between breathing conditions post‐exercise in the competitive swimmers of the present study (Figure 3). However, the higher V̇O_2_, V̇CO_2_, and V̇_E_ under the breath‐holding condition for only the first minute post‐exercise (Figure 4) suggests replenishment of the O_2_ stores used during exercise and expiration of the CO_2_ that accumulated during breath holding, as evidenced by the increased P_et_CO_2_ (Figure 2h), was resolved quickly. In contrast, HR, V̇O_2_, V̇CO_2_, and V̇_E_ were reduced beyond the first minute of recovery in nonswimmers in a previous study (Walsh et al., 2025). This suggests that the residual effects of the breath‐holding response previously observed in nonswimmers (Walsh et al., 2025) were not evident in swimmers and may reflect training adaptations in the swimmers, as trained individuals have a more rapid return of post‐exercise metabolism to resting levels compared to untrained individuals (Børsheim & Bahr, 2003). Moreover, faster recovery from breath holding compared to controls has also been observed in artistic swimmers. The faster recovery was noted by a more rapid return of HR and V̇_E_ to baseline post breath holding, despite having a greater reduction in P_et_O_2_ and an increase in P_et_CO_2_ compared to controls (Alentejano et al., 2012).

## 5 CONCLUSION

When competitive swimmers performed 20 s of high‐intensity simultaneous arm and leg ergometry exercise, with and without breath holding, identical power outputs were sustained for both conditions. There was increased reliance on intrinsic O_2_ stores, but not muscle deoxygenation or anaerobic glycolysis, during exercise while breath holding, although it is unknown if there was an increased contribution from high energy phosphates. Intrinsic O_2_ stores may have been sufficient to meet the aerobic energy demand. Moreover, O_2_ stores were replenished quickly once breathing resumed. While there were signs of an enhanced breath‐holding response in swimmers due to 20 s of high‐intensity simultaneous arm and leg ergometry exercise, compared to nonswimmers in a previous study, the exercise response may have prevailed over the breath‐holding response due to the short duration and high intensity of exercise used in the current study. Further, any potential reduced O_2_ availability due to breath‐holding‐induced peripheral vasoconstriction may have been offset by increased O_2_ delivery suggested by the exercise tachycardia during breath holding. However, whether these land‐based findings would be replicated during swimming is unknown, considering that the breath‐holding response is accentuated when the face is immersed in water (Foster & Sheel, 2005). Future studies of high‐intensity swimming exercise will further elucidate the effects of breath holding on sprint swimming.

## AUTHOR CONTRIBUTIONS

**Jeremy Walsh:** Conceptualization; data curation; formal analysis; funding acquisition; investigation; methodology; project administration; resources; software; supervision; validation; visualization. **James L. Ramsey:** Data curation; investigation; methodology. **Nasimi A. Guluzade:** Data curation; formal analysis; investigation; methodology; software. **Robin Faricier:** Data curation; formal analysis; investigation; methodology; software. **Paul Midgely:** Project administration; resources. **Daniel A. Keir:** Formal analysis; methodology; supervision. **Glen R. Belfry:** Conceptualization; data curation; formal analysis; funding acquisition; investigation; methodology; project administration; resources; software; supervision.

## FUNDING INFORMATION

Jeremy Walsh was supported by a National Sciences and Engineering Research Council of Canada (NSERC) Canada Graduate Scholarship – Doctoral grant (CGS D – 579662‐2023).

## CONFLICT OF INTEREST STATEMENT

No conflicts of interest, financial or otherwise, are declared by the authors. The results of the study are presented clearly, honestly, and without fabrication, falsification, or inappropriate data manipulation.

## Data Availability

The datasets generated during and analyzed during the current study are available in the Open Science Framework repository, https://doi.org/10.17605/OSF.IO/V9UCQ.
